# Initial community response to a novel spatial repellent for malaria prevention in Busia County, Kenya

**DOI:** 10.1186/s12936-025-05452-0

**Published:** 2025-07-04

**Authors:** Sheila Muya Ekodir, Lucy Baker, Julius I. Odero, Jane Klein A. Ikapesi, Moureen Ekisa, Albert Casella, April Monroe, Anna Passaniti, Eric Ochomo, Steven A. Harvey

**Affiliations:** 1 Centre for Global Health Research, Kenya Medical Research Institute, Kisumu, Kenya; 2 Department of International Health, Johns Hopkins Bloomberg School of Public Health, Baltimore, MD USA; 3 Johns Hopkins Center for Communication Programs, Baltimore, MD USA; 4 Vector Group, Liverpool School of Tropical Medicine, Liverpool, UK; 5 The Centre for Infectious and Parasitic Diseases Control Research (CIPDCR), Kisumu, Kenya

**Keywords:** Malaria prevention, Spatial repellent, Community acceptability, Vector control, Trials of Improved Practices (TIPs), Kenya, User perceptions, Mosquito density, Social science research, Qualitative research

## Abstract

**Background:**

Malaria transmission in Africa significantly declined between 2005 and 2015 due to widespread distribution of insecticide-treated nets (ITNs). However, since 2015, transmission has increased due to insecticide resistance and biting at times when people are not using ITNs. Spatial repellents (SRs) may help address these challenges. A double-blinded cluster-randomized controlled trial (cRCT) in Busia County, Kenya, reported that Mosquito Shield™, a transfluthrin-based SR, reduced malaria infections by 33.4% during interim analysis and 32.7% by the end of the study, among children aged 6 months to 10 years. Understanding community responses to SRs is critical for their successful deployment and long-term use. This paper reports the initial community response to MosquitoShield™ as part of the Advancing Evidence for the Global Implementation of Spatial Repellents (AEGIS) project.

**Methods:**

Longitudinal qualitative data were collected from 30 households participating in the cRCT, using modified trials of improved practices (TIPs) to assess participants’ perceptions of MosquitoShield’s utility, efficacy, appearance, and user experience with monthly product replacement. This analysis focuses on initial responses recorded one week and two months post-installation. The data were analysed using thematic coding, with researchers blinded to trial arm assignment.

**Results:**

The participants reported a positive initial response to the SR, with a significant perceived reduction in mosquito density and activity. Some also reported concerns about the product’s effectiveness over time and its comparison with existing mosquito control methods, particularly after first replacement. Participants highlighted their perception that the SR provided continuous protection in contrast with the situational protection offered by ITNs. Improvement suggestions included modified installation methods plus a longer-lasting product that protected more space.

**Conclusion:**

MosquitoShield™ shows potential as a promising malaria prevention tool among communities in Busia County, Kenya. Incorporating user feedback and addressing concerns about product installation, duration, and coverage are crucial for successful implementation. Future research exploring community perceptions, cultural factors and behavioural responses related to long-term acceptability and the impact of SRs on malaria transmission will be crucial to ensure effective SR implementation.

## Background

Long-lasting insecticide-treated nets (ITNs) and indoor residual spraying (IRS) have played crucial roles in reducing malaria transmission across Africa. Between 2000 and 2015, the global malaria incidence dropped by 37%, with cases declining from 262 to 214 million, and the mortality rates falling by 60% from 839,000 to 438,000 [1]. Transmission began to increase again in 2015, however, it was driven by mosquito resistance to insecticides and behavioral adaptations of the vectors [2] as well as transmission at times and places where ITNs cannot not provide adequate protection [3–5]. Achieving the WHO’s target of 90% malaria reduction by 2030 will require innovative and complementary vector control tools [6].

Spatial repellents (SRs) are one such tool. These insecticidal products reduce mosquito-borne disease by inhibiting human-vector contact indoors [7–9]. Mosquito Shield™, developed by SC Johnson, is a transfluthrin-based SR that repels mosquitoes and inhibits their ability to find and feed upon human hosts. It consists of an A4-sized clear plastic sheet that passively releases transfluthrin for 28 days, providing coverage for three square meters of indoor space.

To evaluate Mosquito Shield™ efficacy against malaria infection, the Advancing Evidence for the Global Implementation of Spatial Repellents project (AEGIS) conducted a two-year double-blinded cluster-randomized controlled trial (cRCT) in Busia County, Western Kenya (ClinicalTrials.gov ID NCT04766879). Results showed a 32.7% reduction in malaria cases among children ages 6 months to 10 years in households with Mosquito Shield™ compared to those with an identical-looking untreated plastic sheet [10–12].

In addition to its epidemiological and entomological impact, the successful introduction, uptake, and sustained use of a new vector control product such as Mosquito Shield™ requires community buy-in. This has been demonstrated with regard to ITN use in several African countries [13], IRS acceptance in Ghana [14], and spatial repellent uptake in Cambodia [15]. Understanding what factors influence community and household acceptance of an SR will be crucial to its success [16]. These factors include user-perceived efficacy (independent of epidemiological or entomological measures), user comparisons to existing vector control interventions, and user-preferred product characteristics [17]. AEGIS social science research examined these factors over the two-year efficacy trial.

This paper focuses on initial user assessments of Mosquito Shield™ conducted one week and two months after installation, capturing participants’ immediate reactions and early feedback on the product. These early phases were selected to provide a foundational understanding of user perceptions, including initial impressions and suggested improvements, before trends were analyzed over time. By examining these initial responses, the study establishes a baseline to compare with subsequent data collection phases, offering crucial insights into the early acceptability and feasibility of the intervention without preempting long-term findings. This method provides a more precise understanding of the changes noted in subsequent data collection phases.

## Methods

### Study site

**Fig. 1:**
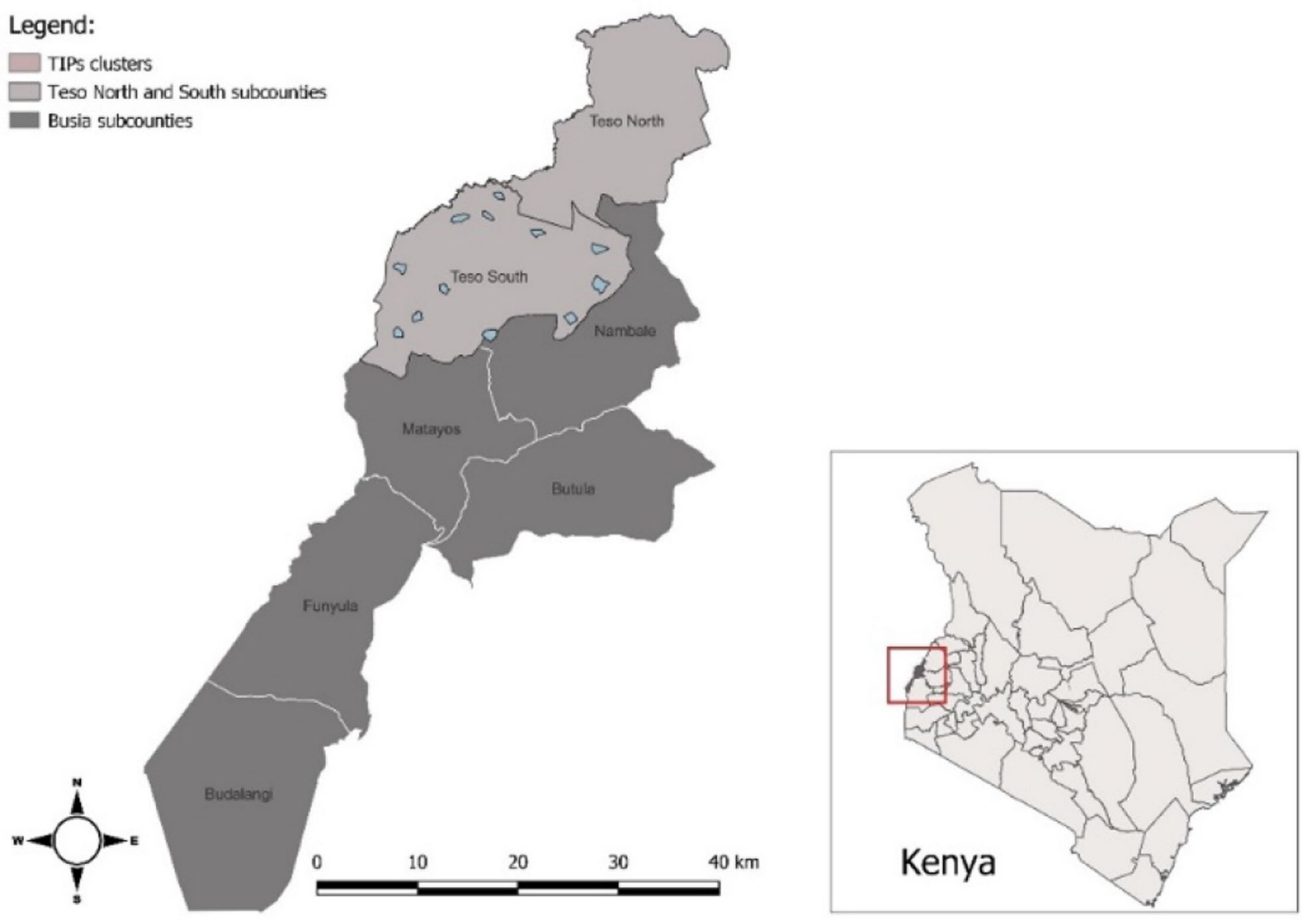
Map of Busia and Subcounties

Busia County, located in Western Kenya, near Uganda border and the northwestern shore of Lake Victoria (Fig. 1). It is a malaria hyperendemic region with year-round transmission. Transmission peaks during the region’s long rainy season (late March to early June) and shorter rains occur from October–November [10]. In June 2021, just prior to the trial’s launch, the National Malaria Control Programme (NMCP) distributed piperonyl-butoxide (PBO) ITNs to all households in the study area [18]. In September 2021, MosquitoShield™ SRs, or identical untreated plastic sheets, were installed in the participating households. Since many rural Kenyan families live in extended households within a single compound, compounds were enrolled, rather than individual households, to better reflect typical living arrangements.

### Sampling

A two-stage sampling to protect the study’s blinded design. First, the study’s independent unblinded statistician randomly selected 12 study clusters, six interventions and six controls. The study team remained blinded to which clusters were intervention and which control. Second, the local community heath promoters (CHPs) who installed the products and also served as volunteers, helped the study team purposively identify two to three compounds within each selected cluster. Purposive sampling was necessary because of the time commitment required for participation in the study’s data collection. The inclusion criteria for compounds were as follows: (1) a verbal commitment to remain in the study for its entire duration (though formally participants still had the right to withdraw if they chose to do so); (2) willingness to participate in 5–6 visits over 24 months; and (3) openness to sharing feedback on the SR, including likes, dislikes, and suggestions for improvement. During installation, CHPs advised participants to avoid close contact with the product and to keep children from touching it.

### Data collection

The Trials of Improved Practices (TIPs) methodology, a participatory research approach developed in the 1980 s to assess health interventions on a small scale before widespread implementation [15]. In AEGIS, TIPs involved five visits over 18 months to a subset of households receiving either the active SR or the placebo. Data was collected from the same households at one week and then two, six, 12, and 18 months after the initial installation. At each TIPs visit, structured observation and then conducted an in-depth interview with an adult compound resident. Owing to the study’s double-blinded design, neither the data collectors nor the participants knew until after the study ended whether any given compound was assigned to intervention or control. The results presented here are based on an analysis of still-blinded data. As a result, the terms “spatial repellent,” “SR,” or “product”, as used in this paper, refer to both active and placebo versions.

During the structured observation, the number of products hanging in each observed room, as well as their appearance, installation method, and any damage, alterations, or loss. Among the compounds with three or more structures, two were selected for observation: one where the head of the compound resided, and either a separate structure used by children or the kitchen if no child-specific structure was present. The data were entered directly into CommCare©, a digital data collection tool [19], using mobile tablet computers. Tablets were also used to photograph any observed anomalies. Following the observation, semistructured qualitative interviews were conducted to explore participants’ and family members’ perceptions of SR effectiveness, side effects, installation issues, improvement suggestions, mosquito density and activity, and malaria transmission. The interviews lasted 30 to 60 min, were audio-recorded, and were conducted in Kiswahili or Ateso, depending on the participant’s preference.

### Data management and analysis

The interview recordings were transcribed verbatim, personal identifiers were removed, and the transcripts were translated into English for analysis. The analysis followed the Sort and Sift, Think and Shift method, which involved continuous, detailed examination of the data, periodic reflection, and iterative adjustment of the analytical approach as needed [20]. This process allowed for the integration of both deductive and inductive insights, ensuring that while the initial codes were guided by predefined research questions, emergent themes were also captured to deepen understanding of community experiences..

Using ATLAS.ti web version 24 [21], two coders independently coded the same transcript and discussed discrepancies to reach consensus. The resulting codes were exported to Microsoft Excel, and grouped by relevant themes: perceived efficacy of the SR, perceived changes in mosquito density and activity; participants’ perceived performance of the SR in relation to other mosquito control methods they commonly use; perception of side effects due to the SR; and suggestions for future improvement of the product. The team held weekly meetings to discuss the progress of data collection, transcription, thematic analysis, coding, and memo-writing. This collaborative process ensured a thorough and reflective analysis of the data, leading to a deeper understanding of the main findings.

## Results

A total of 60 interviews were completed during the first two rounds of data collection, covering 30 households in each round. The participants included 16 women and 14 men. The mean age of the participants was 49 years (range 35–65), with women averaging 44 years (range 35–55) and men 55 years (range 45–65).

In addition to commenting on perceived efficacy, perceived reduction in malaria cases, and side effects, participants compared the perceived performance of SR with other mosquito control methods they commonly used such as bednets, mosquito coils and burning leaves. The participants also discussed their communication with others about the product and suggested future improvements.

### Perceived efficacy and early acceptability

In interviews conducted one week after initial installation, most participants reported that they noticed a reduction in mosquito density and attributed this reduction to the SR’s *dawa* [the power or strength of the insecticide]. Those who observed mosquitoes in the house reported that they appeared sluggish, biting and flying less aggressively than they did in the past.

"*Generally, since you installed these products inside here, I have seen the mosquito population completely reduced. I can say by 99%. Previously, approximately around 7:00 p.m. while seated here [pointing at the sofa set], we would be bitten by mosquitos until when we went to sleep at 9:00 pm. Now, after the installation of the SR, the few mosquitos we see seem sluggish, often flying slowly, and even falling on their own. Additionally, since the installation, I haven’t heard any child complaining about feeling sick.*" (Male, age 39, 1-week post-installation).

In the second interview, 2 months after the initial installation, some participants reported that mosquitoes had returned. They attributed this return to the belief that replacement SRs had less *dawa* than the initial product and lost their effectiveness after 2–3 weeks.

"*How come we are now seeing signs of mosquitos, yet we were praising this product? We are requesting they improve in putting a lot of repellent so that mosquitos do not appear and later disappear. I want, if the product has been installed to repel mosquitos, let it repel mosquitos.*" (Male, age 41, 2-month post-installation).

Some participants also mentioned seasonality, suggesting that the increase in mosquito frequency coincided with millet flowering or the decrease in recent hot weather. One proposed that installing the product during a peak mosquito season could have offered a clearer assessment of its effectiveness.

While most of the comments focused on mosquito density, some respondents added that fewer family members, particularly children, were falling ill with malaria.

"*Since these products were installed in our houses, I have noticed that the number of mosquitos have decreased, and the level of malaria has also decreased. Because before installation, my youngest child had malaria every month, she used to be admitted to the ward. However, since these products were installed, she has never has never been sick.*" (Female, age 28, 2-month post-installation).

In addition to reporting perceived reductions in mosquitoes and malaria symptoms, many participants expressed satisfaction with the SR. They described it as convenient, easy to use, and preferable to other mosquito control tools like nets or coils. These early expressions of acceptance suggest a positive initial reception of the product.

### Comparison of SRs to other mosquito control products

The participants reported using other products and practices to keep away mosquitoes, including ITNs, mosquito coils, mosquito mats, and burning leaves to produce smoke that repels mosquitoes.

When asked to compare the SR to other products, respondents cited various factors including the place of protection, the cost, the product’s perceived effectiveness, and the feasibility of installation. Some mentioned that ITNs only protected them while they were sleeping whereas the SRs also offered protection when they were awake. Others reported that SRs obviated the need for ITNs.

"*I can say that the SR is better than a net because a net is only used when you go to sleep. You only protect yourself with it during sleep. But with the SR, you can be protected while sitting in the evening. Mosquitoes won't bother me because of the SR. However, with a net, you must wait until you go to sleep at 10:00 p.m to be protected.*" (Female, age 32, 1-week post-installation).

"*Because they have installed a product for me that repels mosquitos, I do not see the need to struggle hanging the net because it brings heat. You know during dry season like this there is a lot of heat. There is no need to interfere with the product that repels mosquitos, why not sleep comfortably?*" (Male, age 41, 2-month post-installation).

The respondents also stated that the SR was less labour-intensive than an ITN and that ITNs could tear, make their living spaces hotter, and cause irritation for those who came into direct contact with them.

"*It’s our first time to use spatial repellant, and we are still observing the effects. But within this short time, I see it’s effective and it’s chasing away the mosquitoes even while inside the net, the treated net may lose its effectiveness with time, then the mosquitoes just gain access to you.*" (Male, age 63, 1-week post-installation).

Some added that SRs improved household finances since the study provided them at no cost.

"*The spatial repellent is good because since they were installed in my house, I do not use any money by going to buy other mosquito repellents. But when I did not have [SR], mosquito coils used to cost me money. When you budget for supper, you must put the mosquito coil budget there too.*" (Female, age 28, 1-week post-installation).

### Perceived side effects

While most respondents reported no side effects within their own households, some mentioned observing effects on non-target organisms, including insects such as cockroaches and small animals. One participant described seeing cockroaches that appeared weakened or dead after installation of the SR:

"*What I have experienced with the cockroaches in this house, you find them moving and are weak and some are even dead and others are unconscious there. They are not moving as they used to before and hiding in private places. Currently this product is also affecting them too.*" (Female, age 38, 2-month post installation).

"*Many viewed these effects as an additional benefit of SRs. Some, however, cited conversations in which a neighbor had mentioned side effects such as children sneezing when they got too close to the product or skin irritation experienced by one participant’s husband after touching it. Others expressed uncertainty about whether the effects experienced since installation were directly caused by the SR or by other factors. As a precaution, some participants reported keeping children at a distance from the product to avoid potential adverse effects. Those ‘Ikee’ [Ateso word for medicine or active ingredient, referring to the SR] you are not supposed to get close. At times when you are putting things in order, you need to keep distance from the ‘Ikee’ because we were advised that way. There was a time when my brother’s child who stays here, he got closer to the ‘Ikee’, he started sneezing and I told him that he is not allowed to be close.*" (Female, age 41, 2-month post installation).

### Suggestions for future improvement

At the first TIPs visit, the participants suggested few improvements to the SR, possibly due to their limited experience. Subsequently informants suggested changes in size, shape, colour, smell, installation method, and replacement frequency. A participant suggested alternating the SR’s colour with each replacement not due to aesthetic preference, but to make it easier to recognize whether the product has been changed especially when residents were away during installation.

Other mentioned that white was best because it was visible and matched house décor, while a few said colour did not matter as long as the product worked. Regarding shape and size, some participants suggested enlarging SRs could reduce the number of units required per household. They said they liked that the product was odourless and its general appearance but recommended extending installations to areas such as bathrooms, latrines, and schools where mosquito encounters were common, particularly during the early morning.

"*Then secondly, there are those places these products were not installed. Places like the toilet. If you can find those, then you install. You know you go there anytime, it is dark, and you get bitten. Then again, within here in our home, children study in school. Our children always leave early. Exactly by 6:00 a.m. they are in class. They can get bitten by mosquitoes while there. If it is possible, they should take [SRs] to schools too.*" (Male, 39, 2-month post-installation).

**Fig. 2:**
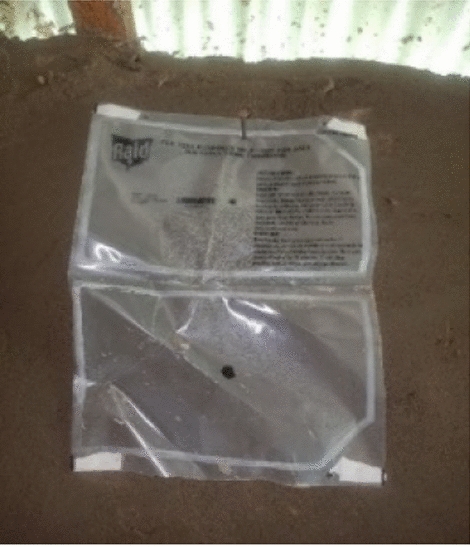
Family rehung fallen taped product with nail

**Fig. 3:**
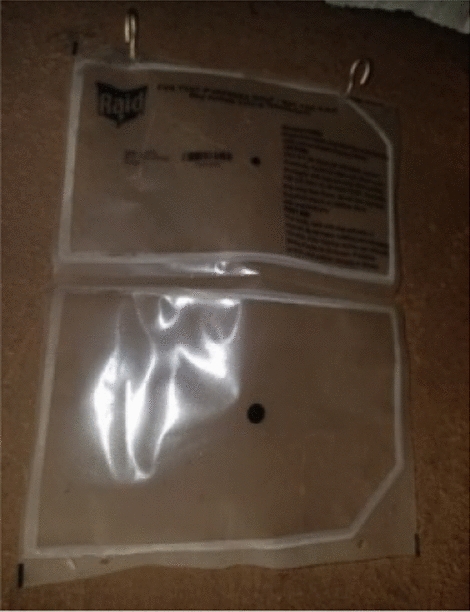
Installation of the MosquitoShield™ with hooks

The participants expressed concerns about the product falling off the wall when installed with tape, which led one family to rehang a fallen product with a nail. They stated their preference for hooks, which kept SRs more securely attached to the wall (Figs. 2 and 3). Some suggested offering a range of colours (black, white, green, blue, khaki) for the products, with one linking a dark colour to attracting mosquitoes.

"*The majority expressed a preference for an odorless product, considering allergies, but a few suggested that some scent was necessary for effective mosquito repellency. A recurring recommendation was to increase the amount or concentration of repellent, with diverse opinions on replacement intervals. Some participants suggested a switch to biodegradable materials to address the environmental concerns associated with plastic sheets.Another one is, we were also saying, they should try and look for a product that is friendly to the environment apart from these papers. At least, even if the project ends and they left the products for us they have not come to collect. At least it should be a material that when you throw, it rots faster.*" (Male, age 39, 2-month post installation).

## Discussion

This study examined the early perceptions of a novel SR among Kenyan participants in a double-blind cRCT testing its epidemiological efficacy. Overall, the majority of respondents initially perceived the SR as effective at reducing both mosquito activity and malaria cases, particularly among children. However, concerns were raised about the consistency of the product’s effectiveness over time, with some suggesting that the presence or amount of active ingredient had declined between the one-week visit and the two-month visit.

While the study was double-blinded, meaning that both participants and research team members were unaware of the study arm assignment, it is possible that placebo effects influenced initial reports of efficacy. The positive feedback received at the one-week visit, especially in the control group, suggests that a placebo effect may have contributed to perceptions of reduced mosquito density and malaria transmission. Once the data are unblinded, the study team will stratify the analysis to determine if reported perceptions about SR correlate with study arm assignment. Meanwhile, the likelihood that some participants perceived a product with no active ingredient to highlights the importance of comparing user perceptions of efficacy with epidemiological and entomological evidence to better assess the true efficacy of the SR.

Some participants recommended expanding SR installation to areas such as schools and latrines, where mosquito encounters are common, particularly in the early morning hours [22]. This is a crucial consideration, as mosquitoes that transmit malaria are often active during these times, and schoolchildren who walk to school in the early morning may be particularly vulnerable. These insights highlight the need for novel vector control measures during times and in places where ITNs and IRS do not offer feasible protection.

Some participants expressed a preference for SRs over other mosquito control methods because of their ease of use and continuous protection. Many reported discontinuing ITN use, stating that the SR alone offered sufficient protection. As a result, the cRCT team revised its communication strategy to emphasize the importance of continued ITN use, especially since SR efficacy had yet to be confirmed and since half the participants were receiving a placebo. Future distributions of SRs should stress the continued importance of continuing to use existing vector control methods since SRs are intended to supplement, not replace, such methods. While participants often expressed a preference for SRs over ITNs due to comfort and perceived ease of use, these responses highlight a potential risk: that communities may abandon foundational tools if SRs are introduced without clear messaging. Effective communication strategies must emphasize that SRs are an additional layer of protection, particularly useful in settings or times where ITNs may fall short, but should be used in combination with, not instead of, existing tools.

Beyond capturing early perceptions, the TIPs process also contributed to community engagement. Through the involvement of the Community Advisory Board (CAB), communication efforts were supported to clarify that SR was not introduced to replace existing malaria tools like ITNs, but rather to complement them. This was particularly important in addressing early misunderstandings and reinforcing continued net use during the study period.

Another factor influencing acceptability is the provision of SRs at no charge. Potential users may view the product more favorably if provided free of cost, while their perceptions might differ if they had to purchase the SR. Further analysis of the full longitudinal dataset will help determine whether these perceptions varied by study arm assignment and whether there was a sustained change in other malaria prevention behaviours such as ITN use. Future programmes should also anticipate a possible need to address concerns about safety, human side effects, and appropriate handling and disposal [23]. Early data suggest a desire for a product that protects a larger area (thus requiring installation of fewer units) and lasts longer (allowing for less frequent replacement).

The participants’ mention of concerns raised by neighbours may reflect social courtesy bias in which individuals attribute their own negative assessments to a third party, fearing that stating such assessments directly might offend study personnel and perhaps threaten their access to products or services provided by or related to the study [24]. However, participants were encouraged to express their honest opinions freely, ensuring that the insights gathered highlight the most important factors influencing product acceptability and sustainability in their specific settings.

These findings align with broader literature on user perceptions of vector control tools, including ITNs, mosquito coils, IRS, and insecticide-treated screens. Across diverse settings, studies have shown that user trust, clarity on product function, and perceived convenience all influence acceptance and sustained use [25–27].These parallels reinforce the value of early TIPs engagement to surface user concerns and preferences prior to large-scale product rollout. Capturing such early perceptions can enhance both the design and messaging of future interventions to better align with community needs and expectations.

## Conclusion

This paper focuses on participants’ initial reactions to MosquitoShield™ and includes data from only the first two of five planned visits. The analysis is based on still-blinded data, so the results are intended to assess initial participant responses to the product in general rather than distinguish between responses on the basis of study assignment. Subsequent analyses will help better understand the long-term community acceptability of the SRs in Kenya.

Future analysis will also benefit from understanding whether and how user perceptions toward the SR changed over time, and whether these perceptions differed on the basis of receiving the active SR or a placebo. Future research should also explore the potential of SRs in combination with other malaria control strategies to optimize their ability to reduce impact on malaria incidence.

## Data Availability

No datasets were generated or analysed during the current study.

## Abbreviations

SR: Spatial repellent
ITN: Insecticide-treated net
IRS: Indoor residual spraying
cRCT: Cluster-randomized controlled trial
AEGIS: Advancing Evidence for the Global Implementation of Spatial Repellents
NMCP: National Malaria Control Programme
TIPs: Trials of improved practices
PBO: Piperonyl butoxide
WHO: World Health Organization
CHPs: Community Health Promoters
